# Factors associated with non-attendance in the Irish national diabetic retinopathy screening programme (INDEAR study report no. 2)

**DOI:** 10.1007/s00592-021-01671-4

**Published:** 2021-01-23

**Authors:** Stephen R. Kelly, Allison R. Loiselle, Rajiv Pandey, Andrew Combes, Colette Murphy, Helen Kavanagh, Patricia Fitzpatrick, Therese Mooney, Patricia Kearney, David P. Crabb, David J. Keegan

**Affiliations:** 1 Mater Retina Research Group, Mater Misericordiae University Hospital, Dublin, Ireland; 2 Department of Ophthalmology, University Medical Centre Groningen, Groningen, Netherlands; 3 Northgate Public Services, NEC, Tokyo, Japan; 4 Diabetic RetinaScreen, National Screening Service, Health Service Executive, Cork, Ireland; 5 Programme Evaluation Unit, National Screening Service, Health Service Executive, Dublin, Ireland; 6 School of Public Health, Physiotherapy and Sports Science, University College Dublin, Dublin, Ireland; 7 Department of Epidemiology, University College Cork, Cork, Ireland; 8 Optometry and Visual Sciences, School of Health Sciences, City, University of London, London, UK

**Keywords:** Diabetic retinopathy, Screening, Public health, Socio-economic issues

## Abstract

**Aims:**

We aimed to determine the patient and screening-level factors that are associated with non-attendance in the Irish National Diabetic Retinal screening programme (Diabetic RetinaScreen). To accomplish this, we modelled a selection of predictors derived from the historical screening records of patients with diabetes.

**Methods:**

In this cohort study, appointment data from the national diabetic retinopathy screening programme (RetinaScreen) were extracted and augmented using publicly available meteorological and geospatial data. A total of 653,969 appointments from 158,655 patients were included for analysis. Mixed-effects models (univariable and multivariable) were used to estimate the influence of several variables on non-attendance to screening appointments.

**Results:**

All variables considered for analysis were statistically significant. Variables of note, with meaningful effect, were age (OR: 1.23 per decade away from 70; 95% CI: [1.22–1.24]), type 2 diabetes (OR: 1.10; 95% CI: [1.06–1.14]) and socio-economic deprivation (OR: 1.12; 95% CI: [1.09–1.16]). A majority (52%) of missed appointments were from patients who had missed three or more appointments.

**Conclusions:**

This study is the first to outline factors that are associated with non-attendance within the Irish national diabetic retinopathy screening service. In particular, when corrected for age and other factors, patients with type 2 diabetes had higher rates of non-attendance. Additionally, this is the first study of any diabetic screening programme to demonstrate that weather may influence attendance. This research provides unique insight to guide the implementation of an optimal and cost-effective intervention strategy to improve attendance.

**Supplementary Information:**

The online version contains supplementary material available at (10.1007/s00592-021-01671-4)

## Introduction

Diabetic retinopathy is a leading cause of vision loss in working-aged and elderly people globally [1]. Many countries have introduced national screening programmes to facilitate the early detection of diabetes-related eye disease [1–3]. While these programmes have proven to be successful in preventing blindness, non-attendance remains an issue.

In Ireland, Diabetic RetinaScreen is a government-funded national screening programme that aims to reduce diabetes-related sight loss, namely from diabetic retinopathy (DR) or diabetic maculopathy. RetinaScreen began in February 2013 and provides annual call–recall screening to persons with diabetes mellitus aged 12 years and over. It is implemented and managed by the National Screening Service (NSS) which is a part of the Health Service Executive (HSE) and is the first non-cancer population to undergo screening by the NSS. In 2019, there were 171,557 people known to have diabetes in Ireland who were eligible to be on the RetinaScreen register. A total of 158,690 people have been invited to attend screening appointments since the inception of the programme [4]. By 2019, there were 147,965 patients who had consented to take part in the programme, with 10,725 (7%) having opted out or been removed from the registry. Non-attendance is an important factor to consider in a DR screening programme because it has been associated with ultimately higher costs and worse vision outcomes in diabetes patients [5–9].

Previous studies of screening programmes in other countries have shown that age, diabetes type, and driving time to the screening location were associated with non-attendance, but with conflicting results depending on the population and study design [1, 5, 10]. The rate of non-attendance and reasons associated with non-attendance has yet to be investigated in Ireland’s RetinaScreen programme, and this is the main idea of this report. Understanding the causes of non-attendance in RetinaScreen could lay the foundation for determining which population subgroups should be targeted for interventions, such as improved communications and alternative screening locations and/or appointment times.

The aim of this study was therefore to determine the patient and screening-level factors that are associated with non-attendance in the Irish national diabetic retinopathy screening programme. To accomplish this, we modelled a selection of predictors derived from the historical screening records of patients with diabetes. Some of these predictors, such as temperature, are novel and have not been included in other studies on non-attendance within diabetic retinopathy screening programmes.

## Methods

### Study population

Patient’s data were extracted from the Diabetic RetinaScreen database used to store details on screening appointments between March 2013 and March 2020. These records included details on patients’ sex, date of birth, address, type of diabetes (type 1 diabetes mellitus; T1DM or type 2 diabetes mellitus; T2DM) as well as details of their screening time, date, and clinic location. The screening programme uses two-field (macula centred and disc centred) 45-degree images captured on quality assured and standardized cameras [4]. All study participants provided informed written consent prior to the study as a part of the screening programme consent form and this study followed the tenets of the Declaration of Helsinki.

### Risk factors

Risk factors used for the models in the current study were as follows: sex, age, diabetes type, socio-economic status (SES), driving time to the clinic, years since the first appointment, previous non-attendance, ambient temperature (binarized above and below freezing or 0 ℃), and a clinic location at an optometric practice. Based on the distribution of non-attendance by age, we determined that age was best represented as decades away from a reference of 70 years old.

Socio-economic data were obtained from the 2016 Pobal HP Deprivation Index. It is a combined score of demographic profile, social class composition, and labour market situation for each Electoral Division (ED) (smallest area of analysis currently available for all Ireland). Weather information was obtained from historical data from the Irish Meteorological Service and contains information on rainfall, temperature and barometric pressure at an hourly resolution from weather towers around the country. The data from the tower nearest the patient at the time of their appointment were included.

Driving times for each appointment were calculated using the Open Source Routing Machine (OSRM) which uses road network mapping from OpenStreetMap. It calculates the distance (in metres) and time (in seconds) between two geographical locations, taking into account the average speed and speed limits of the roads on the journey. Journey times were calculated from the centroid of an ED to the address of the clinic location. Patients that did not have an ED in their file were excluded from the study.

### Data analysis

The predictors were modelled univariably against attendance using a mixed-effects logistic regression model. The predictors were included as fixed effects, and patients were modelled as random effects. Alternative mixed-effects models were tested that included a clinic-level random effect and a nested clinic-patient random effect but were not chosen based on Akaike information criterion (AIC) values.

All analyses were carried out in R (version 4.0.1; R Foundation for Statistical Computing, Vienna, Austria). A p-value of 0.001 or less was considered statistically significant.

## Results

**Fig. 1:**
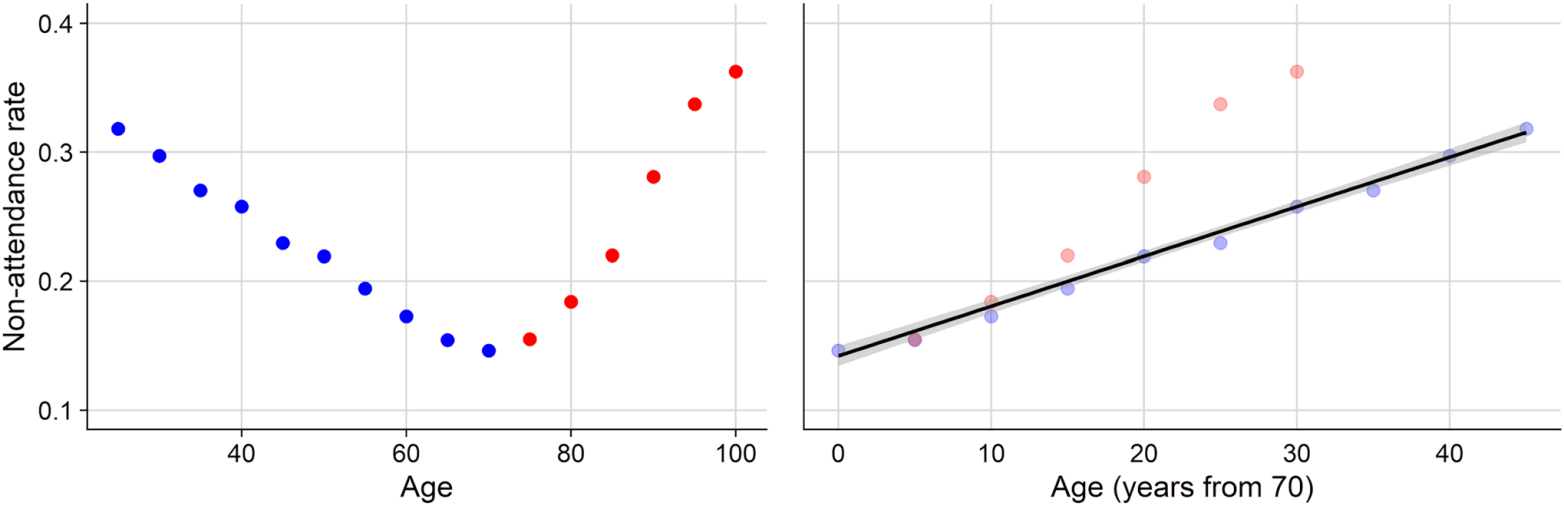
Non-attendance rate by age (left) and non-attendance rate by rate absolute number of years from 70 (right). The blue and red points represent data from those under and over 70 years old, respectively. For the purposes of illustration, data were censored for those younger than 25

A total of 653,969 appointments from 158,655 patients over seven years were included in the analysis. Some patients (1328) were excluded due to missing ED data. An additional 35 patients were excluded due to being labelled as having both T1DM and T2DM. Table 1 depicts the characteristics of the study population. There were 16,988 Type 1 diabetic (T1DM) patients and 141,667 Type 2 diabetic (T2DM) patients. Median (IQR) age was 65.2 (54.6–73.8) years. The overall non-attendance rate for all appointments was 18.5%; and for individual patients, it was 33.9% (i.e., the proportion of patients with at least one missed appointment). Figure 1 shows the distribution of non-attendance by age (left panel). As the relationship is nonlinear, including it in a logistic regression model as a continuous variable would lead to a poor estimation of its influence. We therefore used decades from 70 as a predictor (Fig. 1, right panel), which more closely approximates a linear relationship with non-attendance.

**Table 1:** Characteristics of the study population

	*n* = 158,655
Age (yrs; median [IQR])	65.2 (54.6 to 73.8)
Sex (% Female)	40.2%
Driving time to screening clinic (mins; median [IQR])	14.6 (7.8 to 24.6)
Diabetes Type (% Type 1)	10.7%
Pobal HP Index (deprivation score; median [IQR])	− 5.8 (− 10.7 to -0.4)
At least one missed appointment (% Yes)	37.0%
Years in screening programme (yrs; median [IQR])	3.3 (1.2 to 4.6)
*Repeated non-attendance*	
Percentage of study population that missed three or more appointments (%)	9.6%
Percentage of all missed appointments by patients who missed three or more appointments	52.8%

Table 2 shows the results of the univariable analysis. In these models, female sex, age further away from 70, T1DM, low SES, increased driving time to clinic, increased years since the first appointment, previous non-attendance, freezing temperatures, and clinic appointments at optometrists were associated with a significantly increased risk of non-attendance. Taking age as an example, this can be interpreted as the risk of non-attendance increasing by 24% for each decade the patient is older or younger than 70 years old. For a driving time, a patient with a 60 min commute to the screening clinic is 1.01^3^ = 1.03 or 3% more likely to miss an appointment than someone who has a 30 min commute. Table 3 shows the results of the multivariable logistic regression model. In this model, all of the predictors that were significant in the univariate model were also significant, and the associated risk of non-attendance was in the same direction, except for diabetes type. This can be explained by a younger population in the T1DM cohort compared to T2DM. As age has a larger effect on non-attendance compared to diabetes type, the effect reverses when this is corrected for.

**Table 2:** Results from univariable models

Predictor	Odds ratio	Confidence interval	*p*
Sex [Male]	0.92	0.90–0.94	< 0.001
Age [Decades from 70]	1.24	1.22–1.25	< 0.001
Diabetes Type [T2DM]	0.72	0.70–0.75	< 0.001
Driving Time [per 10 min]	1.01	1.00–1.02	< 0.001
Years Since First Appointment	1.06	1.06–1.07	< 0.001
Previous non-attendance [Yes]	1.50	1.47–1.54	< 0.001
Temperature [Freezing]	1.57	1.45–1.70	< 0.001
Opticians [Yes]	1.55	1.46–1.64	< 0.001
Pobal HP Index Quintiles (reference group is Q3)	–	–	–
Q1 (most deprived)	1.12	1.08–1.16	< 0.001
Q2	1.03	0.99–1.07	0.896
Q4	1.01	0.98–1.05	0.485
Q5 (most affluent)	0.94	0.91–0.97	< 0.001

**Table 3:** Results from multivariable model

Predictor	Odds ratios	Confidence interval	*p*
(Intercept)	0.07	0.07–0.07	< 0.001
Sex [Male]	0.94	0.92—0.96	< 0.001
Age [Decades from 70]	1.23	1.22—1.24	< 0.001
Diabetes Type [T2DM]	1.10	1.06—1.14	< 0.001
Driving Time [per 10 min]	1.01	1.00—1.02	< 0.001
Years Since First Appointment	1.02	1.01—1.02	< 0.001
Previous non-attendance [Yes]	1.52	1.48—1.56	< 0.001
Temperature [Freezing]	1.68	1.55—1.81	< 0.001
Opticians [Yes]	1.66	1.57—1.75	< 0.001
Pobal HP Index Quintiles (reference group is Q3)	–	–	–
Q1 (most deprived)	1.12	1.09—1.16	< 0.001
Q2	1.03	1.00—1.07	0.088
Q4	1.00	0.97—1.04	0.913
Q5 (most affluent)	0.91	0.88—0.94	< 0.001

## Discussion

This study is the first to examine the factors associated with non-attendance within the Diabetic RetinaScreen Programme. We have shown that female sex, age further away from 70 years, T2DM, low SES, longer driving time to the screening clinic, increase in years since the first appointment, previous non-attendance, and a screening appointment located at an optometry practice were all associated with a significantly increased risk of non-attendance. Additionally, this is the first study of any diabetic screening programme to include a novel possible predictor, namely local hourly temperatures. Moreover, a majority of missed appointments (52%) were from patients who had missed three or more appointments, indicating that there may be a subgroup of patients who are unable or unwilling to interact with the screening service. Summary statistics on this subgroup can be found in the Supplementary Materials.

### Screening programmes

Despite a decrease in DR in populations with improved diabetes control, the crude global prevalence of diabetes-related visual impairment and blindness has continued to increase since the 1980s. A meta-analysis of 35 studies estimated that the global prevalence of DR in patients with diabetes was around 34.6% [11]. Screening programmes are, therefore, paramount to mitigating the personal and financial impacts of DR. The benefits of screening for DR are already well known [12, 13]. Visual impairment due to diabetes is lower in countries that have screening programmes [12]. Programmes in countries like England and Wales have reported retinopathy rates of 21% and 30.5%, respectively [14, 15]. While there are no national studies reporting the incidence of DR in Ireland, the GUIDANCE study reported a microvascular complication rate of 26.3% among the Irish participants [16]. Countries that screen for DR also benefit financially, spending 7–20 times less than the cost of supporting those diabetes patients if they became blind [12]. [17] Although the implementation of screening programmes can be cost-effective, national schemes are less common in low and middle-income countries, likely due to the initial cost of setting up such programmes.

In 2016, DR was no longer the leading cause of certifiable blindness in the working-age group in England and Wales thanks to the success of their national DR screening programme, with an uptake of 83% [2]. Diabetic RetinaScreen was established in 2013 and sought to replicate the success of other screening models such as the UK. However, a recent study found that one-third of patients who were eligible to register had not done so [18]. In addition to improving patient uptake, mitigation of non-attendance for those who are already part of the programme will minimize both costs and poor visual outcomes for diabetes patients. It is important to note that a non-attendance rate of 20% reduces the cost-effectiveness of a programme by ~ 10%. [19, 20] The deeper understanding of the specific factors leading patients to miss appointments that were found in this study could yield a more targeted and efficient intervention approach. This model has been deployed effectively in an Irish context already, and we will look to expand on an international basis. [18, 21]

### Age and non-attendance

In the univariable analysis, patients with T1DM seemed to have higher non-attendance rates but, once corrected for age and other variables in the multivariable model, this effect was reversed. This is likely due to younger people, who have higher rates of non-attendance in general, making up a higher proportion of the T1DM population. Figure 1 (left panel) depicts a bimodal distribution of non-attendance rates by age, showing that it is the youngest and the oldest populations that are more likely to miss appointments. A London-based study found a similar U-shaped non-attendance rate with the youngest and oldest populations missing more appointments [22]. Many studies, however, only reported younger age being associated with higher non-attendance rates [5]. Two studies from south-east London and Tayside, Scotland, respectively, found that non-attendance rates were higher in younger patients [23, 24]. A third study in Ireland also found that younger age was associated with higher non-attendance, however, the sample size was small and the methodology of univariable logistic regression makes it difficult to draw strong conclusions [25]. In all of these studies, the relationship between age and attendance is either treated as linear or stratified into age groups. We suggest that due to the underlying bimodal (U-shaped) relationship between non-attendance and age, including age in a model in these ways skews the observed effect depending on the underlying population being studied. In these examples, a population whose age is mostly under 70 would show younger age being associated with increased non-attendance. In contrast, a study looking at the screening uptake among T2DMs in Germany between 2004 and 2013 found that it was older patients and those with disabilities who had lower rates of screening. The youngest age group in this study was 50–69. In this instance, the lack of data on younger patients skewed the direction of the observed effect towards older patients [26]. In our analysis, we included a transformed age variable to correct for the U-shaped relationship between non-attendance and age (Fig. 1).

### SES and non-attendance

In the current study, we found an association between socio-economic deprivation and higher rates of non-attendance. The Pobal HP Index is a combined summary measure of various subscores based on socio-economic factors of people residing in a geographical area. We found that when compared to a reference group (the third/middle quintile), the most deprived quintile had around 12% higher non-attendance rates. The most affluent quintile conversely had a 9% lower non-attendance rate. These findings are echoed in much of the literature. When comparing the first and fifth quintiles only, we found that the first quintile had a 21% higher non-attendance rate. A review on non-attendance within DR screening found that socio-economic deprivation was associated with higher non-attendance rates in all studies that reported it [10]. In London, one study found a difference in uptake by SES but this was not reflected in any difference in sight-threatening DR. [22] Another study in Gloucestershire similarly found that uptake was lower in socioeconomically deprived populations but unlike the London study, sight-threatening DR was also associated with SES [27]. In the aforementioned Tayside study, those in the most deprived quintile were 2.3 times more likely to miss a screening appointment compared to those in the most affluent quintile [24]. In south east London, attendance rates were significantly lower in patients residing in areas with the highest levels of deprivation. [23] In a qualitative study looking at the influence of primary care practices on the uptake in DR screening, deprivation was a factor listed as a barrier to uptake [28]. The negative relationship between socio-economic deprivation and health is well-documented and not limited to diabetes. It is mainly driven by a combination of material, psychosocial and behavioural factors that lead to higher rates of morbidity in socioeconomically deprived groups [29]. Given the high rates of private health cover in Ireland (46%), we had estimated that there may have been a bimodal non-attendance peak for screening with the least affluent not attending for the above reasons but also with the most affluent, as they may choose to attend a private practitioner over the free state-funded scheme [30]. Indeed many patients were attending DR screening privately before the introduction of the national programme and have continued to do so. However, the predicted bimodal distribution of non-attendance does not appear to be the case in our diabetic population. Once registered with the national screening service, the most affluent quintile missed less appointments when compared to more deprived quintiles.

### Travel time and non-attendance

We found a small increase in non-attendance with an increase in driving time to the screening clinic (OR: 1.01 per 10 min). It is possible that there is a difference between urban and rural settings where public transport is relied on more heavily. An Irish study looking at attendance at treatment centres after referral from the national DR screening programme found that people living over 60 km away were less likely to attend [31]. The above-mentioned review by Kashim et al. noted conflicting findings on whether distance to the clinic was associated with non-attendance [10]. In the Tayside study, travel time was not associated with non-attendance but in a study by Lindenmeyer and colleagues in England, it was [24, 28]. It is likely that the effect of travel time on attendance depends greatly on the demographics of the underlying population and the availability of different types of transport. A secondary analysis of our data showed a slightly higher rate of non-attendance for patients living outside of the capital city Dublin compared to those living in Dublin (18.9% non-attendance and 17.3% non-attendance, respectively).

### Non-attendance and poor outcomes

Non-attendance to screening appointments is associated with worse visual outcomes for patients with diabetes. [6–8] If patients are not screened, it increases the likelihood that early retinal lesions will not be detected. A study from Iceland looked at past screening attendance rates of people registered on their national blindness registry. Those registered as blind had a worse attendance record hinting at an association between non-attendance and vision impairment. [6] One study found that the patient attendance rates to ophthalmic clinics were lower among those who were registered as partially sighted or blind [7]. The situation is more complex than simply attending or not attending screening appointments and there are many possible confounding factors at play. Patients who miss appointments are also the ones that tend to have poorer overall health. A systematic review listed myriad poor health outcomes associated with non-attendance, including microvascular (retinopathy and neuropathy) and macrovascular (coronary artery disease and peripheral vascular disease) complications [5]. One study looked at the change in DR in three London boroughs and found that patients who missed screening for one and two years were more likely (OR: 3.4 and 10.8, respectively) to have referable retinopathy [8]. A later study from the same group found a lower incidence of sight-threatening DR from patients with mild nonproliferative retinopathy or no retinopathy at their first visit [32]. They argued that patients at low risk may be suited for less frequent (for example, biennial) screenings.

### Interventions

A systematic review of the effectiveness of interventions to increase uptake in DR screening programmes found that all but one study in 48 showed an increase in attendance within the intervention group [33]. Another review concluded that essentially any intervention targeting patients, providers, or the health care system was associated with a meaningful increase in DR screening compared to usual care [34]. In fact, there was no difference between DR screening specific interventions and a general quality improvement of diabetes care strategy [34]. A study of uptake in DR screening found that integrating the screening with other care, such as routine diabetes care or a seasonal flu vaccine, also increased attendance [28].

A previous study in Ireland found that one of the main barriers to receiving adequate screening was a lack of knowledge regarding the need for ocular examination and that a previous physician recommendation about the necessity of a regular eye examination improved attendance [35]. Others found that improving patient and clinician awareness of DR screening was effective [33]. The Diabetic RetinaScreeen programme has evolved the information and communication approaches over the six rounds of screening to improve this, and uptake rates have generally improved. [4]

In the current study, we found that non-attendance was more frequent when screenings took place at optometric practices. This effect was present for both chain and independent optometrists. This could be an issue with the perception of the role of optometrists. It is possible that patients associate optometrists with visual complaints, and if they do not have visual symptoms from their diabetes, they do not see the need for an optometrist. Conveying the importance of DR-specific ocular examinations is essential, and optometrists in Ireland play a role for one of the contracted providers for the Diabetic RetinaScreen programme, in particular in locations outside Dublin. In addition, optometrists engage patients about their visual health and can act as a valuable reinforcer of the need for regular attendance at Diabetic RetinaScreen.

A funded study in Ireland, soon to be underway, aims to improve uptake in the national DR screening service by introducing provider and patient-level interventions; provider interventions in the form of training, audit, feedback, and reimbursement and patient interventions in the form of a GP-endorsed reminder, an information leaflet and a phone/letter reminder [36]. It’s likely that this will increase uptake but to what degree remains to be seen.

Lastly, it is important to consider inter-individual variation in reasons for non-attendance. One qualitative study found differences in attitudes and motivational factors in screening attendance between younger adults and older adults. Additionally, in the current study, the number of years in the programme was associated with an increase in non-attendance. This could mean that patients who have many appointments with no visual deterioration place less importance on screening. These patients may be candidates for a reminder of the importance of screening, even after years of attendance. A successful and cost-effective intervention needs to be tailored towards its intended patient population and not have a one-size-fits-all approach [37].

### Strengths and limitations

The main limitation of this study was the lack of available information on HbA1C values. Other studies have highlighted the association between high non-attendance rates and poor glycaemic control, and it would have been of interest to replicate those findings [5]. Although there is an association between non-attendance and poorer outcomes (discussed below) without associated HbA1c values, we cannot make any associations between non-attendance and glycaemic control. There was also no visual acuity information in this dataset. A comparison of visual outcomes with attendance would have been useful and is being incorporated into the Optomize™ programme which is the core Electronic Medical Record of Diabetic RetinaScreen. One major strength of this study is the large and diverse sample size. As a national database, the patients included were from all over Ireland and represented a diverse range of demographic profiles. Another strength of this study was using a more statistically sound approach of a mixed-effects model with a transformation of age. Finally, by linking the patients’ appointments with historical weather data, we were able to demonstrate a relationship between non-attendance and below-zero (freezing) temperatures, likely due to the presence of ice on the roads, which has not previously been shown. In this study, only 0.8% of the total appointments were on days where the temperature was below freezing. However, we believe this may be especially relevant to consider programmes implemented in other countries with colder climates.

This study outlines factors that are associated with non-attendance within the Irish national diabetic retinopathy screening service. Some of the factors listed give insight into which population subgroups may benefit from a targeted intervention.

## Supplementary Information

 Supplementary file 1 (DOCX 6 kb)

## References

[1] Ting DSW, Cheung GCM, Wong TY (2016). Diabetic retinopathy: global prevalence, major risk factors, screening practices and public health challenges: a review. Clin Exp Ophthalmol.

[2] Scanlon PH (2017). The english national screening programme for diabetic retinopathy 2003–2016. Acta Diabetol.

[3] Gangwani RA, Lian JX, McGhee SM (2016). Diabetic retinopathy screening: global and local perspective. Hong Kong Med J.

[4] Pandey R, Morgan MM, Murphy C (2020). Irish National Diabetic RetinaScreen Programme: report on five rounds of retinopathy screening and screen-positive referrals (INDEAR study report no 1). Br J Ophthalmol.

[5] Brewster S, Bartholomew J, Holt RIG, Price H (2020). Non-attendance at diabetes outpatient appointments: a systematic review. Diabet Med.

[6] Zoega GM, Gunnarsdóttir T, Björnsdóttir S (2005). Screening compliance and visual outcome in diabetes. Acta Ophthalmol Scand.

[7] Rhatigan MC, Leese GP, Ellis J (1999). Blindness in patients with diabetes who have been screened for eye disease. Eye.

[8] Forster AS, Forbes A, Dodhia H (2013). Non-attendance at diabetic eye screening and risk of sight-threatening diabetic retinopathy: a population-based cohort study. Diabetologia.

[9] Waqar S, Bullen G, Chant S (2012). Cost implications, deprivation and geodemographic segmentation analysis of non-attenders (DNA) in an established diabetic retinopathy screening programme. Diabetes Metab Syndr.

[10] Kashim RM, Newton P, Ojo O (2018). Diabetic retinopathy screening: a systematic review on patients’ non-attendance. Int J Environ Res Pub Health.

[11] Yau JWY, Rogers SL, Kawasaki R (2012). Global prevalence and major risk factors of diabetic retinopathy. Diabetes Care.

[12] Stefánsson E, Bek T, Porta M (2000). Screening and prevention of diabetic blindness. Acta Ophthalmol Scand.

[13] Jones S, Edwards RT (2010). Diabetic retinopathy screening: a systematic review of the economic evidence. Diabet Med.

[14] Jones CD, Greenwood RH, Misra A, Bachmann MO (2012). Incidence and progression of diabetic retinopathy during 17 years of a population-based screening program in England. Diabetes Care.

[15] Thomas RL, Dunstan F, Luzio SD (2012). Incidence of diabetic retinopathy in people with type 2 diabetes mellitus attending the diabetic retinopathy screening service for wales: retrospective analysis. BMJ.

[16] Stone MA, Charpentier G, Doggen K (2013). Quality of care of people with type 2 diabetes in eight European countries: findings from the guideline adherence to enhance care (GUIDANCE) study. Diabetes Care.

[17] Vujosevic S, Aldington SJ, Silva P (2020). Screening for diabetic retinopathy: new perspectives and challenges. Lancet Diabetes Endocrinol.

[18] Tracey M, Racine E, Riordan F (2019). Understanding the uptake of a national retinopathy screening programme: an audit of patients with diabetes in two large primary care centres. HRB Open Res.

[19] Broadbent DM, Sampson CJ, Wang A (2019). Individualised screening for diabetic retinopathy: the ISDR study-rationale, design and methodology for a randomised controlled trial comparing annual and individualised risk-based variable-interval screening. BMJ Open.

[20] Harding SP, Sampson CJ, Criddle TM (2019). The costs of screening for sight-threatening diabetic retinopathy. Invest Ophthalmol Vis Sci.

[21] Riordan F, Racine E, Phillip ET (2020). Development of an intervention to facilitate implementation and uptake of diabetic retinopathy screening. Implement Sci.

[22] Gulliford MC, Dodhia H, Chamley M (2010). Socio-economic and ethnic inequalities in diabetes retinal screening. Diabet Med.

[23] Millett C, Dodhia H (2006). Diabetes retinopathy screening: audit of equity in participation and selected outcomes in South East London. J Med Screen.

[24] Leese GP, Boyle P, Feng Z (2008). Screening uptake in a well-established diabetic retinopathy screening program: the role of geographical access and deprivation. Diabetes Care.

[25] Bennett GH, Tuthill A (2017). Investigating the barriers to the uptake of Diabetic RetinaScreen. Ir Med J.

[26] Kreft D, McGuinness MB, Doblhammer G, Finger RP (2018). Diabetic retinopathy screening in incident diabetes mellitus type 2 in Germany between 2004 and 2013 - A prospective cohort study based on health claims data. PLoS ONE.

[27] Scanlon PH, Carter SC, Foy C (2008). Diabetic retinopathy and socioeconomic deprivation in Gloucestershire. J Med Screen.

[28] Lindenmeyer A, Sturt JA, Hipwell A (2014). Influence of primary care practices on patients’ uptake of diabetic retinopathy screening: a qualitative case study. Br J Gen Pract.

[29] Arcaya MC, Arcaya AL, Subramanian SV (2015). Inequalities in health: definitions, concepts, and theories. Glob Health Action.

[30] (2020) Market figures for patients insured with inpatient health insurance. Health Insurance Authority. https://www.hia.ie/publication/market-statistics, https://www.hia.ie/sites/default/files/Market%20Figures%20June%202020.pdf. Accessed 10 Sept 2020

[31] Greenan E, Salim M, Coakley DN, James M (2019). The effect of geodemographic factors on the attendance rates at a regional diabetic retinopathy treatment centre. Ir J Med Sci.

[32] Forster AS, Forbes A, Dodhia H (2013). Changes in detection of retinopathy in type 2 diabetes in the first 4 years of a population-based diabetic eye screening program: retrospective cohort study. Diabetes Care.

[33] Zhang X, Norris SL, Saadine J (2007). Effectiveness of interventions to promote screening for diabetic retinopathy. Am J Prev Med.

[34] Lawrenson JG, Graham-Rowe E, Lorencatto F (2018). Interventions to increase attendance for diabetic retinopathy screening. Cochrane Database Syst Rev.

[35] Dervan E, Lillis D, Flynn L (2008). Factors that influence the patient uptake of diabetic retinopathy screening. Ir J Med Sci.

[36] Riordan F, Racine E, Smith SM (2020). Feasibility of an implementation intervention to increase attendance at diabetic retinopathy screening: protocol for a cluster randomised pilot trial. Pilot Feasibility Stud.

[37] Lake AJ, Browne JL, Rees G, Speight J (2017). What factors influence uptake of retinal screening among young adults with type 2 diabetes? A qualitative study informed by the theoretical domains framework. J Diabetes Complicat.

