# Supplementary material for: Factors associated with non-attendance in the Irish national diabetic retinopathy screening programme (INDEAR study report no. 2)
Source: Acta Diabetol. 2021 Jan 23;58(5):643–50. doi: 10.1007/s00592-021-01671-4 (PMC8076137; doi:10.1007/s00592-021-01671-4)
Supplement: Supplementary file 1 — (DOCX 6 kb) [file 592_2021_1671_MOESM1_ESM.docx]

**Supplementary Materials:**

**Table A: Summary statistics on the subgroup of patients that missed three or more appointments.**

|  | **n=15,293** |
| --- | --- |
| Age (yrs; median [IQR]) | 59.8 (47.8 to 71.1) |
| Sex (% Female) | 42.2% |
| Driving time to screening clinic (mins; median [IQR]) | 15.1 (7.3 to 26.5) |
| Diabetes Type (% Type 1) | 14.1% |
| Pobal HP Index (deprivation score; median [IQR]) | -6.2 (-11.3 to -0.7) |
| At least four missed appointment (% Yes) | 55.6% |
| At least five missed appointment (% Yes) | 29.7% |
| Years in screening programme (yrs; median [IQR]) | 4.5 (3.5 to 5.1) * |

* It should be noted that most of these patients will be in the screening programme for at least three years to have been able to miss three appointments.
